# OGT regulates RNA-binding protein nucleocytoplasmic partitioning, but this fails in amyotrophic lateral sclerosis

**DOI:** 10.1016/j.isci.2026.117807

**Published:** 2026-10-08

**Authors:** Yiran Wang, Lisa Fournier, Benjamin Wymann, Kevin Talbot, Raphaëlle Luisier, Rickie Patani

**Affiliations:** 1 Department of Neuromuscular Diseases, Queen Square Institute of Neurology, University College London, London, UK; 2 The Francis Crick Institute, 1 Midland Road, London, UK; 3 Oxford Motor Neuron Disease Centre, Nuffield Department of Clinical Neurosciences, University of Oxford, John Radcliffe Hospital, Oxford, UK; 4 Kavli Institute for Nanoscience Discovery, University of Oxford, Dorothy Crowfoot Hodgkin Building, Oxford, UK; 5 Department for BioMedical Research, University of Bern, Bern, Switzerland; 6 Swiss Institute of Bioinformatics, Lausanne, Switzerland; 7 Department of Oncology, Hôpitaux Universitaires de Genève, Geneva, Switzerland; 8 Neurobiology Programme, Life Sciences Institute, Centre for Life Sciences, National University of Singapore, Singapore, Singapore; 9 Department of Medicine, Yong Loo Lin School of Medicine, National University of Singapore, Singapore, Singapore; 10 Department of Anatomy, Yong Loo Lin School of Medicine, National University of Singapore, Singapore, Singapore; 11 Neuroscience Translational Research Programme, Yong Loo Lin School of Medicine, National University of Singapore, Singapore, Singapore

**Keywords:** O-GlcNAcylation, OGT, OGA, RNA-binding protein, nucleocytoplasmic localization, amyotrophic lateral sclerosis

## Abstract

O-linked N-acetylglucosamine transferase (OGT) catalyzes the O-linked β-N-acetylglucosamine (O-GlcNAc) modification to proteins and has been implicated in a range of diseases, including amyotrophic lateral sclerosis (ALS), a rapidly progressive and currently incurable neurodegenerative disorder. However, the precise mechanisms by which OGT influences ALS pathogenesis remain largely unresolved. In this study, we used human-induced pluripotent stem cell (hiPSC)-derived spinal motor neurons (hiPSC-MNs), the principal cell type that undergoes degeneration in ALS, to systematically reveal that OGT-related mechanisms regulate RNA-binding protein (RBP) nucleocytoplasmic compartmentalization. Using a combination of antisense oligonucleotide (ASO) technology and small molecular pharmacology, we reveal two distinct regulatory mechanisms of RBP compartmentalization, including by intron-retaining transcript-mediated sequestration and O-linked N-acetylglucosaminylation (O-GlcNAcylation)-mediated nuclear transport. We finally examine the tractability of manipulating OGT activity in the ALS context but demonstrate that the OGT-related mechanisms governing RBP localization in healthy hiPSC-MNs are impaired in the context of ALS-causing *VCP* and *TARDBP* mutations.

## Highlights

• *OGT* intron-retaining transcript interacts with multiple RNA-binding proteins
• Reducing *OGT* intron retention promotes nuclear localization of bound RBPs
• Increasing O-GlcNAcylation independently promotes nuclear RBP localization
• OGT-mediated RBP localization is impaired in ALS patient-derived motor neurons

## Graphical abstract

**Figure:**
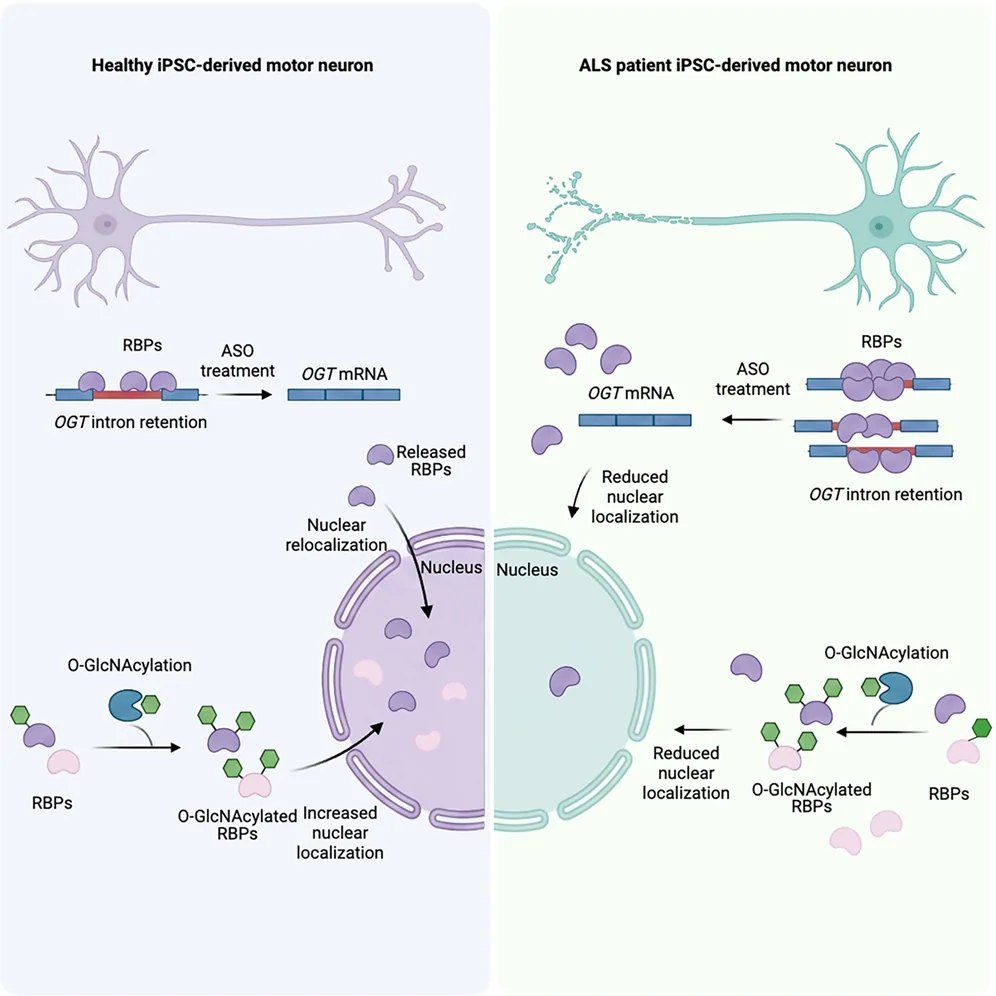

## Teaser

Molecular biology; Neuroscience; Cell biology; Stem cells research

## Introduction

O-linked N-acetylglucosaminylation (O-GlcNAcylation) is a prevalent and crucial post-translational modification (PTM) that links serine (Ser) and threonine (Thr) residues in proteins to O-linked β-N-acetylglucosamine (O-GlcNAc).^1^ This PTM in turn influences protein activity,^2^ stability,^3^^,^^4^ and interactions.^5^^,^^6^ The enzyme O-linked N-acetylglucosamine transferase (OGT) catalyzes the O-GlcNAc modification to proteins^7^^,^^8^ and another enzyme, O-GlcNAc hydrolase (OGA), has the opposite effect.^9^^,^^10^ Both OGT and OGA are ubiquitously expressed, with the highest expression levels in the central nervous system.^11^^,^^12^ Deregulation of O-GlcNAcylation is associated with numerous human diseases ranging from neurodegeneration to cancer.^13^^,^^14^^,^^15^^,^^16^

Amyotrophic lateral sclerosis (ALS) is a rapidly progressive and invariably fatal neurodegenerative disease characterized by the degeneration of motor neurons in both the brain and spinal cord, leading to loss of essential functions in patients, such as moving, speaking, swallowing, and respiration.^17^^,^^18^ A hallmark of ALS pathology is the aberrant nucleocytoplasmic distribution of RNA-binding proteins (RBPs), including the nuclear-to-cytoplasmic (NC) mislocalization of transactive response (TAR) DNA-binding protein 43 (TDP-43), fused in sarcoma (FUS), and splicing factor proline- and glutamine-rich (SFPQ), consistently observed across affected neurons in different models.^19^^,^^20^^,^^21^ Through RNA recognition domains, these ALS-associated RBPs have high binding affinity for RNA targets and form a densely interconnected network enriched in RNA metabolism functions, contributing to widespread dysregulation throughout the RNA life cycle in ALS.^22^

O-GlcNAcylation has been increasingly implicated in ALS pathology. Initial studies identified significantly reduced O-GlcNAc levels in spinal motor neurons of SOD1-mutant rodent models, particularly on the neurofilament medium (NF-M) subunit.^23^^,^^24^ Elevating O-GlcNAcylation increased NF-M modification in control mice but not in ALS models, thereby indicating a disrupted regulatory mechanism.^24^ In parallel, aging-dependent oxidative stress and motor neuron loss have been reported to be accompanied by reduced spinal cord O-GlcNAcylation in ALS-like mouse models lacking non-selenocysteine-containing phospholipid hydroperoxide glutathione peroxidase (NPGPx), an oxidative stress sensor. Notably, restoring O-GlcNAc levels alleviated neuronal loss and oxidative damage, highlighting its neuroprotective potential.^25^ More recently, O-GlcNAcylation of TDP-43, specifically at amino acids T199 and T233, was shown to reduce the hyperphosphorylation and pathological aggregation of TDP-43 and restore its splicing activity on downstream transcripts, such as *Stathmin-2* and *Sortlin1*, underscoring the function of OGT and O-GlcNAcylation in modulating ALS-related molecular dysfunction.^26^ Aberrant intron retention, a form of pre-mRNA alternative splicing where introns are retained in mature mRNAs, in *OGT* transcripts with highly cytoplasmic abundance has been reported during motor neurogenesis in induced pluripotent stem cell (iPSC) models from ALS patients carrying valosin-containing protein (*VCP*) mutations.^27^ Furthermore, the same study showed that *OGT* retained intron 4 possesses sequence-specific and high predicted binding affinity to several RBPs.^27^ More recently, a study using modification-specific enrichment and multiplexed proteomics in a non-ALS human cancer cell line MCF7 suggested that O-GlcNAcylated proteins have overall higher nuclear distributions compared with corresponding non-modified ones.^28^ Given that TDP-43, FUS, and SFPQ are known to undergo nuclear-to-cytoplasmic mislocalization in ALS-affected neurons and that O-GlcNAcylated proteins exhibit overall higher nuclear distributions in non-neuronal cells,^28^ we hypothesized that O-GlcNAcylation may play a direct role in maintaining the nuclear localization of ALS-relevant RBPs in motor neurons.

Here, we used both antisense oligonucleotide (ASO) technology and small molecular pharmacology to study the subcellular compartmentalization of the RBPs either bound to the *OGT* retained intron 4 or known for nuclear-to-cytoplasmic mislocalization in ALS-affected neurons. We reveal two distinct regulatory mechanisms of RBP nucleocytoplasmic distribution via targeting the *OGT* retained intron 4 and through OGA inhibition in healthy human iPSC-derived motor neurons (hiPSC-MNs). However, these regulatory mechanisms are disrupted in ALS hiPSC-MNs. These findings provide mechanistic insights into the widespread mislocalization of RBPs observed in ALS.^22^

## Results

### Targeting *OGT* intron retention in human motor neurons using an ASO

We hypothesized that aberrant intron 4 retention in *OGT* may regulate the nucleocytoplasmic distribution of RBPs, which is based on our previous study where we found that *OGT* intron-retaining transcript (IRT) is highly enriched in the cytoplasm, and that RBPs bound to the intronic sequence may be partitioned to the cytoplasmic compartment.^27^ In order to test this hypothesis, we leveraged an optimized splice-switching ASO targeting a decoy splice site within intron 4, which acts by engaging intron-terminal splice sites and competing with cross-intron interactions.^29^ Using our established protocol for generating hiPSC-MNs, we transfected an ASO targeting the decoy 5′ splice site, or a non-targeting fluorescein amidite (FAM)-labeled ASO in different concentrations as transfection indicators. Fluorescence-activated cell-sorting analysis showed approximately 50% transfection efficiency across concentrations (Figure 1A). To evaluate the effects of ASO on regulating *OGT* intron retention, we performed qPCR and established that ASO-OGT treatment dose-dependently reduces the levels of IRT (Figure 1B). We went on to use 75 nM ASO in all further experiments, as it significantly reduces *OGT* IRT, while inducing a reciprocal increase in *OGT* protein-coding transcript and protein expression without causing overt elevation of a stress marker, Activating Transcription Factor 3 (*ATF3*), in cells^30^ (Figures 1C–1F).

**Figure 1:**
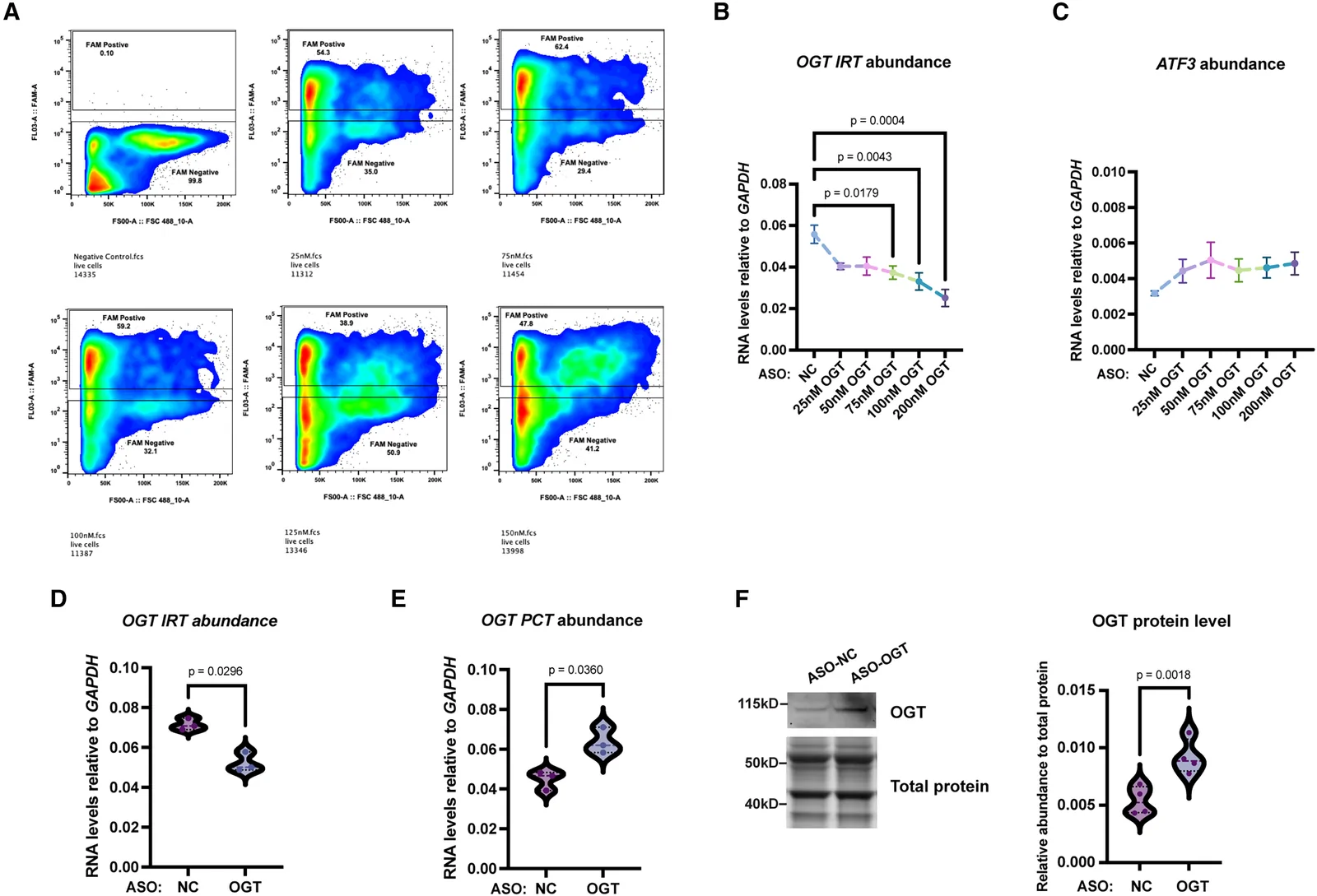
ASO targeting *OGT*-aberrant intron 4 in hiPSC-derived motor neurons (A) Fluorescence-activated cell-sorting (FACS) analysis of FAM-labeled ASO treatments in different concentrations. (B) Dose-dependent effect of ASO-OGT on downregulating *OGT* IRT. *p* values were determined from the one-way ANOVA and Dunnett’s multiple comparisons test following the Shapiro-Wilk normality test, *n* = 3 hiPSC-MN lines, across two independent experimental repeats. (C) Stress marker *ATF3* levels with different concentrations of ASO-OGT. Statistical analysis was performed using the one-way ANOVA following the Shapiro-Wilk normality test; no significant treatment effect was detected. *n* = 3 hiPSC-MN lines. (D) The effect of ASO-OGT on *OGT* IRT at 75 nM. *p* value was determined from the paired two-tailed *t* test following the Shapiro-Wilk normality test, *n* = 3 hiPSC-MN lines. (E) The effect of ASO-OGT on *OGT* protein-coding transcript at 75 nM. *p* value was determined from the paired two-tailed *t* test following the Shapiro-Wilk normality test, *n* = 3 hiPSC-MN lines. (F) The effect of ASO-OGT on OGT protein expression. *p* value was determined from the paired two-tailed *t* test following the Shapiro-Wilk normality test, *n* = 4 hiPSC-MN lines. Data are presented as mean ± SEM or violin plots.

### Decreasing *OGT* intron retention induces an increase in the nuclear-to-cytoplasmic ratio of RBPs in healthy human motor neurons

We previously examined the RBP profiles associated with *OGT* retained intron 4 by exploring the ENCODE cross-linking and immunoprecipitation (CLIP) data for 112 RBPs in K562 and HepG2 cells.^31^^,^^32^ Ranking these proteins by the proportion of cross-linking events in the *OGT* retained intron 4 revealed polypyrimidine tract-binding protein 1 (PTBP1), U2 small nuclear RNA auxiliary factor 65 kDa subunit (U2AF65), heterogeneous nuclear ribonucleoprotein K (hnRNPK), and U2 small nuclear RNA auxiliary factor 35 kDa subunit (U2AF35), among the most highly enriched RBPs.^27^ We next investigated the localization of predicted RBP candidates of *OGT* IRT and other ALS-related mislocalized RBPs (TDP-43, FUS, and SFPQ) following ASO-OGT treatment, which we also observed bind to *OGT* retained intron 4 using ENCODE CLIP analysis (Figure 2A). We found that downregulation of the *OGT* IRT results in a significant increase in the nuclear-to-cytoplasmic ratio of 3 out of 4 RBPs highly bound to *OGT* intron 4, including PTBP1, U2AF65, and hnRNPK, but not to TDP-43, FUS, SFPQ, or U2AF35 (Figures 2B–2H). These data raise two (non-mutually exclusive) possibilities: (1) the nucleocytoplasmic localization of PTBP1, U2AF65, and hnRNPK is determined by *OGT* intron 4, or (2) the increase in OGT caused by the ASO that decreased *OGT* intron retention affects the subcellular distribution of these RBPs by O-GlcNAcylation.

**Figure 2:**
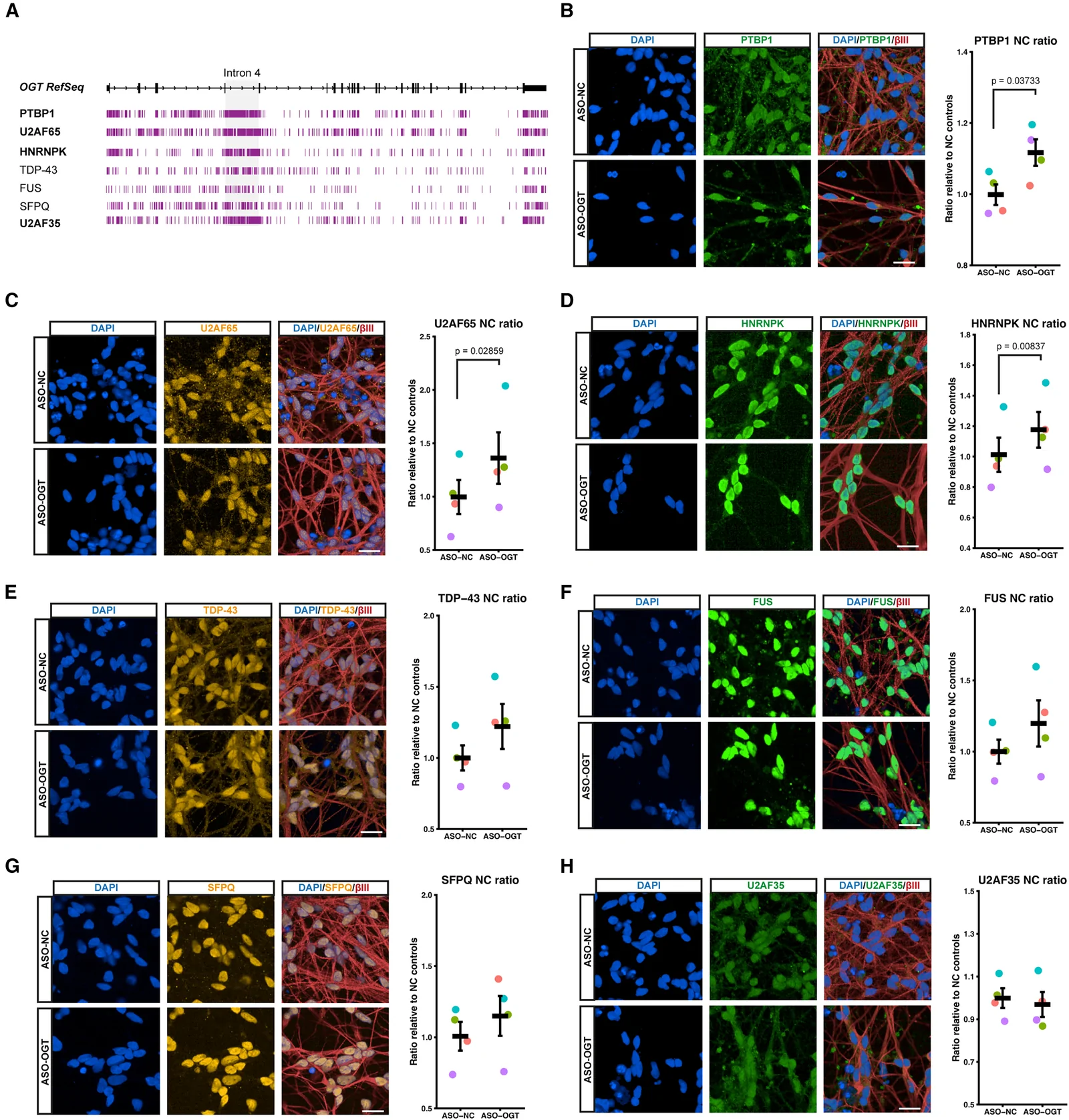
RNA-binding protein localization after ASO treatment targeting *OGT* retained intron 4 in healthy control hiPSC-derived motor neurons (A) Genome browser view of PTBP1, U2AF65, hnRNPK, TDP-43, FUS, SFPQ, and U2AF35 CLIP cross-linking events along *OGT* transcript annotations. Gray box highlights the location of the retained intron 4. (B–H) Nucleocytoplasmic localization assay for RBPs: PTBP1 (B), U2AF65 (C), hnRNPK (D), TDP-43 (E), FUS (F), SFPQ (G), and U2AF35 (H). *n* = 4 healthy hiPSC-MN lines, across three to five independent experimental repeats. Each data point represents the mean for one cell line across experimental repeats, with values calculated relative to the ASO-NC group mean within each repeat (CTRL1, orange; CTRL2, green; CTRL3, blue; CTRL4, purple). *p* values were determined from the paired two-tailed *t* test following the Shapiro-Wilk normality test. Data are presented as scatterplots with mean ± SEM. Scale bars: 20 μm.

### Pharmacologically enhancing O-GlcNAcylation increases nuclear-to-cytoplasmic ratio of RBPs in healthy human motor neurons

We next explored orthogonal means of manipulating O-GlcNAcylation. Thiamet G (TMG) is an inhibitor of OGA, the enzyme that removes O-GlcNAc. Therefore, TMG increases O-GlcNAcylation levels by inhibiting OGA and is functionally analogous to our splice-switching ASO used above, which ultimately acts to increase *OGT* protein and promotes the attachment of O-GlcNAc to its target proteins. We first examined the global O-GlcNAc modification in hiPSC-MNs comparing ASO-OGT versus TMG treatments and found that TMG robustly increased global O-GlcNAcylation, compared to the relatively low concentration (75 nM) of ASO targeting *OGT* retained intron 4 (Figure S1). This suggests that the effect of ASO-OGT on nucleocytoplasmic distribution of RBPs is primarily due to the elimination of *OGT* IRT and the potential consequent release of RBPs. Against this background, we employed TMG and assessed the nucleocytoplasmic distributions of each of the aforementioned RBPs. TMG treatment results in a significant increase in the nuclear-to-cytoplasmic ratio of U2AF65, hnRNPK, TDP-43, FUS, and SFPQ but not PTBP1 or U2AF35 (Figures 3A–3G). Taken together, these data suggest that OGT-mediated O-GlcNAcylation contributes to the subcellular localization of U2AF65, hnRNPK, TDP-43, FUS, and SFPQ. This does not exclude a possible concurrent contribution from the cytoplasmic *OGT* intron 4-retaining transcript. These data also suggest that the nucleocytoplasmic distribution of PTBP1 is likely regulated primarily by its binding to cytoplasmic *OGT* intron 4-retaining transcript.

**Figure 3:**
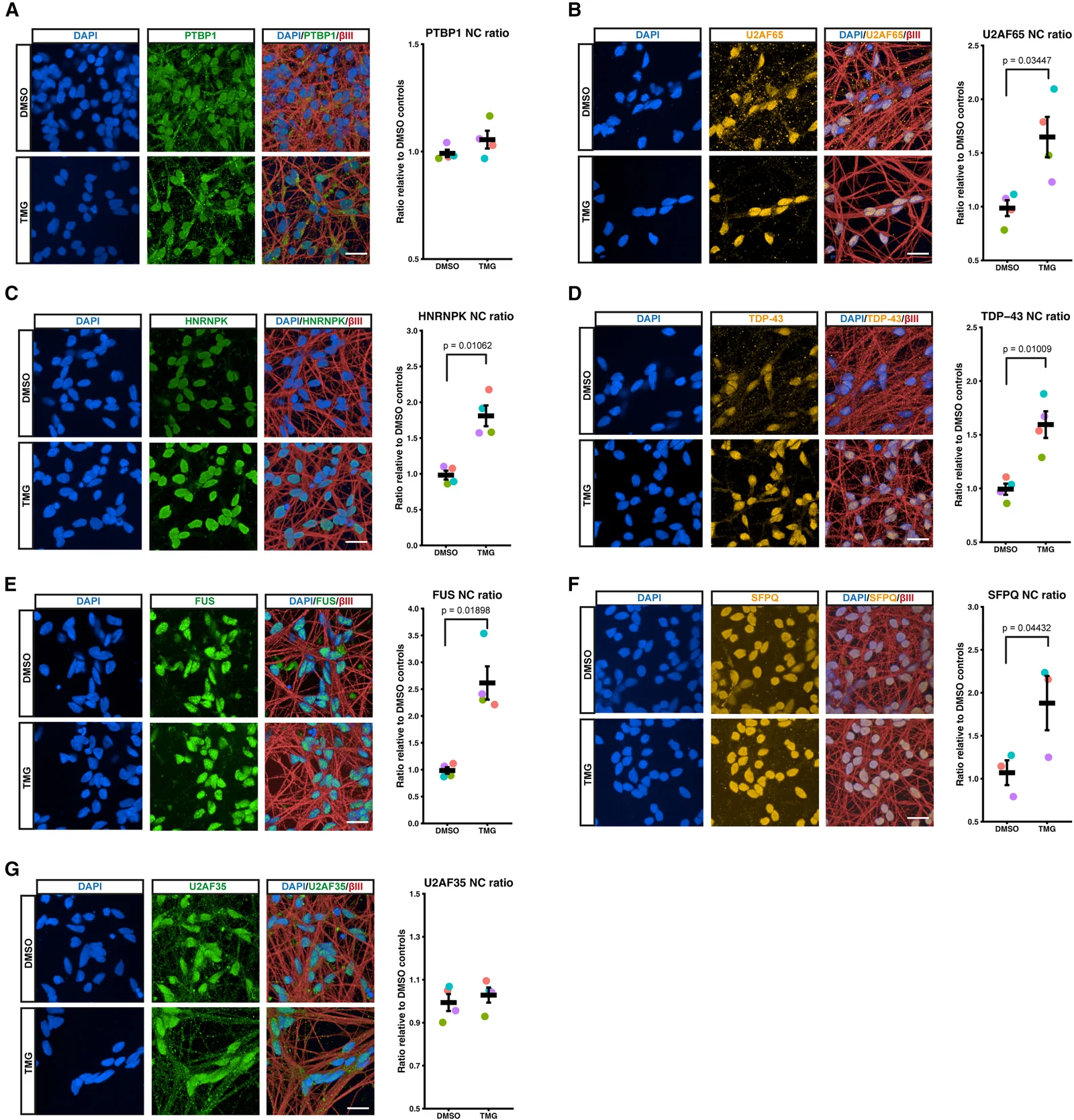
RNA-binding protein localization after OGA inhibition in healthy control hiPSC-derived motor neurons Nucleocytoplasmic localization assay for RBPs: PTBP1 (A), U2AF65 (B), hnRNPK (C), TDP-43 (D), FUS (E), SFPQ (F), and U2AF35 (G). *n* = 3–4 healthy hiPSC-MN lines, across two to four independent experimental repeats. Each data point represents the mean for one cell line across experimental repeats, with values calculated relative to the DMSO group mean within each repeat (CTRL1, orange; CTRL2, green; CTRL3, blue; CTRL4, purple). *p* values were determined from the paired two-tailed *t* test following the Shapiro-Wilk normality test. Data are presented as scatterplots with mean ± SEM. Scale bars: 20 μm.

### OGT-mediated regulation of RBP nucleocytoplasmic distribution is perturbed in ALS

Noting our findings so far that OGT plays roles in regulating nucleocytoplasmic localization of RBPs in healthy cells, we next addressed whether targeting OGT is a tractable therapeutic strategy in ALS, where RBPs have been reported to undergo mislocalization mainly from the nucleus to the cytoplasm. We therefore studied the impact of the splice-switching ASO targeting *OGT* intron 4 and TMG treatments. In contrast to its ability to increase nuclear localization in healthy motor neurons, the ASO failed to achieve a similar effect in motor neurons carrying ALS-causing mutations in the *VCP* gene (*VCP*^*R155C*^ and *VCP*^*R191Q*^). Specifically, there was no impact on PTBP1, hnRNPK, TDP-43, FUS, SFPQ, and U2AF35 (Figures 4A and 4C–4G). However, somewhat surprisingly, U2AF65 exhibited a significant increase in the nuclear-to-cytoplasmic ratio following treatment of *VCP* ALS motor neurons with the ASO targeting *OGT* intron 4 (Figure 4B). Treatment of *VCP* ALS motor neurons with TMG also failed to increase nuclear-to-cytoplasmic ratio of these RBPs (Figure S2). Specifically, there was no significant change in PTBP1, hnRNPK, TDP-43, FUS, and SFPQ. Unexpectedly, there was a significant decrease in the nuclear-to-cytoplasmic ratio of U2AF65 and U2AF35. Using the exhaustively curated database of experimentally identified O-GlcNAc-modified proteins, O-GlcNAcAtlas,^33^^,^^34^ which contains over 4,400 human proteins and displays detailed modification sites, the majority of RBPs in our study are modified, except for U2AF35/U2AF65. The O-GlcNAcylation on U2AF35/U2AF65 may have not been reported, or may not occur at all, which would explain why these proteins behave differently in our experiments compared to other RBPs.

**Figure 4:**
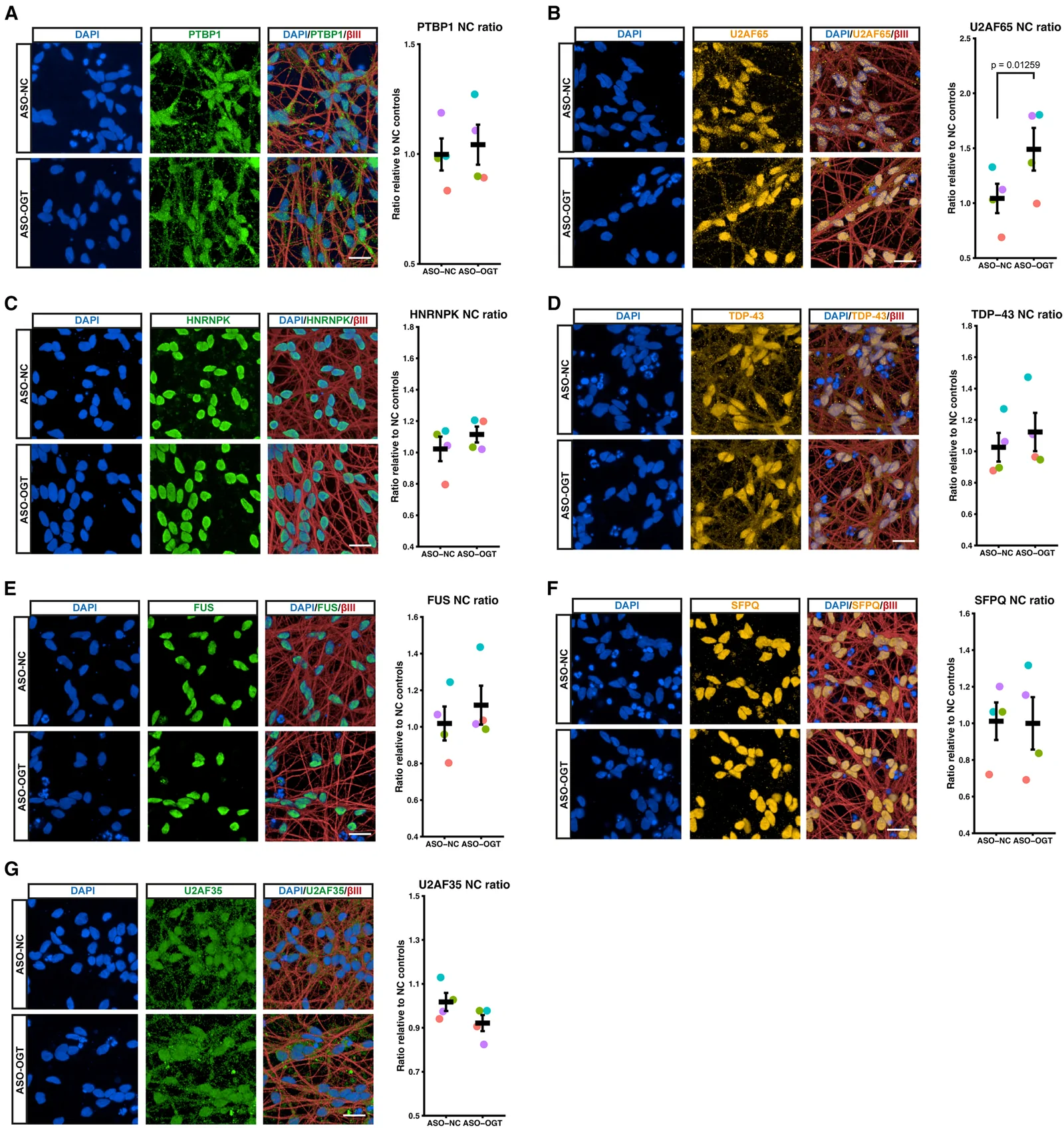
RNA-binding protein localization after ASO treatment targeting *OGT* retained intron 4 in ALS-*VCP* mutant hiPSC-derived motor neurons Nucleocytoplasmic localization assay for RBPs: PTBP1 (A), U2AF65 (B), hnRNPK (C), TDP-43 (D), FUS (E), SFPQ (F), and U2AF35 (G). *n* = 4 ALS-*VCP* hiPSC-MN lines, across two to four independent experimental repeats. Each data point represents the mean for one cell line across experimental repeats, with values calculated relative to the ASO-NC group mean within each repeat (VCPmut1, orange; VCPmut2, green; VCPmut3, blue; VCPmut4, purple). *p* values were determined from the paired two-tailed *t* test following the Shapiro-Wilk normality test. Data are presented as scatterplots with mean ± SEM. Scale bars: 20 μm.

Finally, we examined the effect of ASO-OGT or TMG on the nucleocytoplasmic distribution of RBPs in ALS patient lines carrying the *TARDBP*^*G298S*^ mutation. We found that ASO-OGT or TMG does not affect subcellular localization of these proteins in *TARDBP* mutant hiPSC-MNs, similar to the OGT-related deregulation in the *VCP* mutation model (Figure S3). Altogether, these data demonstrate that the regulatory role of OGT in RBP nucleocytoplasmic partitioning becomes perturbed in the context of *VCP*- and *TARDBP*-related ALS.

## Discussion

The nucleocytoplasmic partitioning of RBPs is regulated by a complex interplay of specific factors (e.g., nuclear importins, exportins, and transport-partners) and processes (e.g., transcription rate, nucleocytoplasmic distribution of RNAs and their stability, and PTMs to RBPs).^35^^,^^36^ An example of a PTM regulating RBP localization comes from arginine methyltransferase inhibitors, which have been shown to ameliorate the cytoplasmic mislocalization of FUS.^37^ A relatively understudied PTM in the context of RBP localization and ALS is O-GlcNAcylation. Here, we established the role of OGT as a regulator of RBP nucleocytoplasmic localization through two different mechanisms. The first mechanism involves promoting *OGT* intron 4 splicing, which leads to more *OGT* protein-coding transcript that is available for translation to *OGT* protein. Increased OGT levels may directly lead to increased O-GlcNAcylation of specific RBPs, which in turn may regulate their subcellular distribution. The second (but not necessarily mutually exclusive) mechanism is that intron 4, when retained, sequesters—or at least increases the residency time of—different RBPs to the specific cellular compartment it occupies. We have previously demonstrated that *OGT* IRT is abundant in the cytoplasm, which would argue that the RBPs that are bound with the highest occupancy to it are also rendered cytoplasmic in their distribution.^27^ It follows that decreasing *OGT* IRT may lead to relocalization of several RBPs to the nucleus, as we demonstrated here for PTBP1, U2AF65, and hnRNPK. We also employed a potent strategy to manipulate O-GlcNAcylation using TMG, an inhibitor of OGA. OGA inhibition increased the nuclear-to-cytoplasmic ratio of hnRNPK, U2AF65, TDP-43, FUS, and SFPQ.

It is noteworthy that decreasing *OGT* retained intron 4 using an ASO was sufficient to significantly increase the nucleocytoplasmic ratio of 3 out of the 7 tested RBPs, which are among the most enriched RBPs binding to *OGT* intron 4 based on our previous analysis of ENCODE CLIP datasets,^27^ without altering the global O-GlcNAcylation in motor neurons. Also, OGA inhibition using TMG had a significant effect on 5 out of these 7 RBPs. PTBP1 is only increased upon decreased *OGT* intron retention but not altered by TMG, and this may suggest that PTBP1 nucleocytoplasmic localization is regulated predominantly by its binding to the *OGT* retained intron 4. U2AF65 and hnRNPK are regulated by both mechanisms, while TDP-43, FUS, and SFPQ nucleocytoplasmic partitioning is regulated, at least in part, by O-GlcNAcylation rather than potential binding to *OGT* intron 4. Increased O-GlcNAcylation of TDP-43 was associated with increased nuclear localization, in line with previous findings suggesting that O-GlcNAcylation may compete with TDP-43 phosphorylation and modulate its downstream function in neuronal cell models.^26^ We recently demonstrated that SFPQ nucleocytoplasmic distribution can be regulated through an alternative splicing event that gives rise to a predominantly cytoplasmic isoform.^38^ Notably, U2AF35 seems not to be significantly regulated by either of the aforementioned OGT-related mechanisms in normal physiology.

Having examined the ability of OGT to regulate nucleocytoplasmic partitioning of RBPs in healthy motor neurons, we next turned our attention to its capacity in a disease context, using ALS as an example. Specifically, we previously demonstrated that a large number of IRTs exhibit a specific increase in cytoplasmic abundance in *VCP* mutant cultures.^27^ This suggests that *VCP* mutations lead to aberrant nuclear export and/or cytoplasmic stabilization of a specific class of IRTs, potentially underlying stereotyped cellular dysfunction as a consequence. Examining publicly available CLIP data has shown increased binding of RBPs across a cytoplasmically localized retained intron,^21^ later biochemically confirmed by RNA immunoprecipitation (RIP).^27^ In a subsequent study, we subclassified IRTs spatiotemporally during motor neurogenesis, where we identified a major class of cytoplasmic IRTs that shows a high capacity for RBP binding. In this study, we also showed that ALS-causing *VCP* mutations lead to a selective increase in cytoplasmic abundance of this particular class of IRTs.^39^ We next showed increased interaction between mislocalized RNAs (including IRTs) and RBPs more generally in ALS using *in silico* predictions as well as validation from examining all available public CLIP data.^22^ We examined the stability and regulation of these transcripts in the cytoplasm (including *SFPQ* and *OGT*) in a recent study.^38^

In contrast to what we found in healthy motor neurons, manipulation of *OGT* intron 4 or OGA inhibition showed no significant effects on the nucleocytoplasmic distribution of hnRNPK, PTBP1, TDP-43, FUS, and SFPQ in *VCP* ALS motor neurons. The ASO-decreased *OGT* intron 4-retaining transcript did significantly increase the nuclear-to-cytoplasmic ratio of U2AF65 but had no such effect on the other 6 RBPs tested. OGA inhibition using TMG had the opposite effect on U2AF65 and U2AF35, thereby significantly decreasing their nuclear-to-cytoplasmic ratios. The divergent effects of OGT manipulation between healthy and ALS motor neurons suggest that the *VCP* mutations impair the ability of OGT to regulate nucleocytoplasmic compartmentalization of RBPs. Interestingly, we have previously shown that 349 transcripts in the same model exhibit significant intron retention, 112 of which undergo some export to the cytoplasm. Of these 112 IRTs, 49 are predominantly partitioned to the cytoplasm.^27^ It is therefore possible that targeting *OGT* intron 4 or inhibiting OGA failed to relocalize mislocalized RBPs because of the high cytoplasmic burden of IRTs that we have demonstrated in the disease context. In this scenario, mislocalized RBPs may bind to any of the multitude of cytoplasmic IRTs that contain the corresponding motifs, and targeting only one of the 112 transcripts may be insufficient to allow therapeutic redistribution of most RBPs. Nevertheless, the strategy of targeting an IRT did indeed permit the relocalization of one of the studied RBPs (U2AF65). This suggests that targeting a cytoplasmic IRT to achieve nuclear relocalization of a bound RBP is potentially a viable strategy, although this will take a similar effort to the current study for robust evaluation of any given target.

In our study, U2AF35 did not exhibit significant changes in nucleocytoplasmic distribution in healthy motor neurons following either ASO-OGT or TMG treatment, which suggests that its transport is unlikely to be directly regulated by *OGT* IRT or the associated PTM under basal conditions. A similar lack of response to ASO treatment was observed in *VCP* mutant motor neurons, further supporting this interpretation. Notably, although U2AF65 demonstrated robust nuclear relocalization following both ASO-OGT and TMG treatments, experimentally confirmed O-GlcNAc modification sites for U2AF65 are not currently cataloged in O-GlcNAcAtlas.^33^^,^^34^ This does not exclude the possibility of O-GlcNAcylation at novel or lowly abundant sites or that U2AF65 may be regulated indirectly via protein-protein interactions with O-GlcNAcylated partners, which merits further investigation. However, in mutant neurons treated with TMG, we observed a shift in the nucleocytoplasmic distribution of several RBPs, including U2AF35 and U2AF65, in the opposite direction to that seen under other conditions. This divergent response indicates that TMG may exert mutation-specific effects on RBP dynamics in subcellular compartments. Although the precise mechanism remains to be elucidated, we speculate that this effect may be associated with ATPase hyperactivity driven by the *VCP* mutations, leading to nuclear pore complex protein mislocalization^22^ and aberrant degradation.^40^ In parallel, OGA inhibitor-mediated upregulation of global O-GlcNAcylation may further alter the regulatory landscape. Together, these changes could collectively disrupt the nucleocytoplasmic transport of a subset of RBPs involved in pre-mRNA splicing.

We also examined the RBP localization changes in *TARDBP* mutant ALS models and observed a loss of regulatory effects of ASO-OGT or TMG on RBP nucleocytoplasmic distribution, similar to that in *VCP* mutant motor neurons. The *TARDBP*^*G298S*^ mutation used in this study is located in the C-terminal low-complexity domain of TDP-43, close to the confirmed O-GlcNAcylation site at T233.^26^ It is therefore conceivable that this mutation directly disrupts the local protein conformation in a region associated with O-GlcNAc-mediated regulation of nuclear localization. The concordance between *VCP* and *TARDBP* mutant neurons suggests that impaired OGT-mediated regulation of RBP localization may be a more general feature of ALS pathology, independent of the causative mutation. As the current study has focused on mutations in the *VCP* and *TARDBP* genes, an important avenue of future research is to examine these phenomena across other ALS mutations including in *C9orf72*, *SOD1*, and *FUS.*

### Limitations of the study

In this study, although we demonstrate the correlation between OGT/OGA manipulation and RBP localization changes, further mechanistic studies beyond the scope of the present work will be important to determine the contribution of direct O-GlcNAc modification of individual RBPs, through the identification of specific O-GlcNAc sites in neuronal systems by mass spectrometry, followed by functional analysis using modification-incompatible mutants (e.g., Ser/Thr to alanine [Ala] substitutions at confirmed sites, particularly those proximal to the nuclear localization/export signal [NLS/NES] sequences). Similarly, identifying the nuclear transport factors responsible for the treatment-induced RBP distribution changes in healthy and ALS motor neurons will provide further insight into the underlying transport mechanisms. Finally, the number of *TARDBP* mutant lines available for this study was modest, and additional independent lines carrying mutations at different positions relative to the TDP-43 O-GlcNAcylation sites^26^ and NLS^41^^,^^42^^,^^43^ will be valuable for further validation and generalization of these findings.

## Resource availability

### Lead contact

Requests for further information and reagents may be directed to and will be fulfilled by the lead contact, Rickie Patani (rickie.patani@nus.edu.sg).

### Materials availability

This study did not generate any new unique materials or reagents.

### Data and code availability

• Original western blot images have been deposited at Mendeley Data: https://doi.org/10.17632/854xtzypt4.1, and are publicly available as of the date of publication. The accession number is also listed in the key resources table.
• The study does not report original code.
• Any additional information required to reanalyze the data reported in this paper is available from the lead contact upon request.

## Acknowledgments

We wish to thank the patients for fibroblast donation. The graphical abstract for this study was created in BioRender. WANG, Y. (2026) https://BioRender.com/u0v1qkq. We are grateful for the technical support provided by the Cell Science, Flow Cytometry, and Screening and Automated Science facilities at the 10.13039/100010438Francis Crick Institute and the Sir William Dunn School of Pathology at the 10.13039/501100000769University of Oxford. Y.W. is funded by a Non-Clinical Junior Research Fellowship from the 10.13039/501100000406Motor Neurone Disease Association (Wang/Oct23/2324). R.P. holds a Lister Research Prize Fellowship and gratefully acknowledges generous support from Steve Redgwell and Liane Iles, MND Foundation, National University of Singapore (NUS), the 10.13039/501100000406Motor Neurone Disease Association (Patani/Dec22/957-793), My Name’5 Doddie Foundation (MN5DF/2022/003), and 10.13039/100013013Target ALS (BB-2024-C4-L4). 10.13039/100010438The Francis Crick Institute receives its core funding from 10.13039/501100000289Cancer Research UK (FC010110), the 10.13039/501100000265UK Medical Research Council (FC010110), and the 10.13039/100010269Wellcome Trust (FC010110).

## Author contributions

Conceptualization, Y.W. and R.P.; formal analysis, Y.W., L.F., and R.L.; methodology, Y.W., L.F., B.W., and R.L.; investigation, Y.W., L.F., R.L., and R.P.; data curation, Y.W., L.F., and R.L.; resources, K.T. and R.P.; funding acquisition, Y.W., K.T., and R.P.; project administration, Y.W. and R.P.; supervision, R.P.; writing – original draft, Y.W. and R.P.; writing – review and editing, Y.W., L.F., B.W., K.T., R.L., and R.P.

## Declaration of interests

The authors declare no competing interests.

## STAR★Methods

### Key resources table

**Table:** 

REAGENT or RESOURCE	SOURCE	IDENTIFIER
**Antibodies**		

Mouse monoclonal anti-PTBP1	Proteintech	Cat#67462-1-Ig; RRID: AB_2882692
Rabbit polyclonal anti-PTBP1	Proteintech	Cat#12582-1-AP; RRID: AB_2176402
Rabbit polyclonal anti-U2AF65	Abcam	Cat#ab37530; RRID: AB_883336
Mouse monoclonal anti-hnRNPK	Santa Cruz	Cat#sc-28380; RRID: AB_627734
Rabbit polyclonal anti-TDP-43	Proteintech	Cat#12892-1-AP; RRID: AB_2200505
Mouse monoclonal anti-FUS	Santa Cruz	Cat#sc-47711; RRID: AB_2105208
Rabbit polyclonal anti-SFPQ	Abcam	Cat#ab38148; RRID: AB_945424
Mouse monoclonal anti-U2AF35	Proteintech	Cat#60289-1-Ig; RRID: AB_2858277
Chicken polyclonal anti-beta III Tubulin	Abcam	Cat#ab41489; RRID: AB_727049
Mouse monoclonal anti-OGT	Santa Cruz	Cat#sc-74546; RRID: AB_2157067
Mouse monoclonal anti-*O*-GlcNAc RL2	Abcam	Cat#ab2739; RRID: AB_303264

**Chemicals, peptides, and recombinant proteins**		

Essential 8 Medium	Gibco	Cat#A1517001
Geltrex	Gibco	Cat#A1413302
UltraPure EDTA	Invitrogen	Cat#15575020
DMEM/F12	Gibco	Cat#31330038
Neurobasal Medium	Gibco	Cat#12348017
*N*-2 Supplement	Gibco	Cat#17502001
B27 Supplement	Gibco	Cat#12587001
GlutaMAX Supplement	Thermo Scientific	Cat#35050038
MEM Non-Essential Amino Acids	Gibco	Cat#11140050
β-Mercaptoethanol	Gibco	Cat#21985023
Human insulin	Sigma-Aldrich	Cat#I9278
Dorsomorphin	Tocris	Cat#3093
SB431542	Tocris	Cat#1614
CHIR99021	Tocris	Cat#4423
Retinoic Acid	Sigma-Aldrich	Cat#R2625
Purmorphamine	Sigma-Aldrich	Cat#540220
Compound E	Tocris	Cat#6476
Lipofectamine RNAiMAX Transfection Reagent	Invitrogen	Cat#13778150
SYBR Green Mastermix	Applied Biosystems	Cat#4309155
Tris-HCl Buffer, pH 7.5	Invitrogen	Cat#15567027
NaCl	Invitrogen	Cat#AM9760G
NP-40 Detergent Solution	Thermo Scientific	Cat#85124
EDTA	Invitrogen	Cat#AM9260G
cOmplete Protease Inhibitor Cocktail	Roche	Cat#4693159001
NuPAGE Reducing Agent	Invitrogen	Cat#NP0009
NuPAGE LDS Sample Buffer	Invitrogen	Cat#NP0007
NuPAGE MOPS SDS Running Buffer	Invitrogen	Cat#NP0001
Paraformaldehyde	Thermo Scientific	Cat#043368.9M
PBS	Gibco	Cat#10010015
Bovine Serum Albumin	Sigma-Aldrich	Cat#A7906
Triton X-100	Thermo Scientific	Cat#9002-93-1
Thiamet G	Sigma-Aldrich	Cat#SML0244

**Critical commercial assays**		

Maxwell RSC simplyRNA Cells Kit	Promega	Cat#AS1390
SuperScript IV First-Strand Synthesis System	Invitrogen	Cat#18091050
Pierce BCA Protein Assay Kit	Thermo Scientific	Cat#23227
Revert Total Protein Stain Kit	LICORbio	Cat#926-11016
ECL Western Blotting Detection Reagent	Cytiva	Cat#RPN2232

**Deposited data**		

Existing RBP CLIP data	ENCODE	https://www.encodeproject.org/
Original Western blot images	This paper	Mendeley Data: https://doi.org/10.17632/854xtzypt4.1

**Experimental models: Cell lines**		

Human: CTRL1 iPSC line	Thermo Fisher Scientific	Cat#A18945
Human: CTRL2 iPSC line	Ludtmann et al.^44^ (Wray lab)	N/A
Human: CTRL3 iPSC line	Cedars-Sinai	CS0002iCTR-nxx; RRID: CVCL_E8CP
Human: CTRL4 iPSC line	NIH CRM	CRMi003-A, RRID: CVCL_1E71
Human: VCPmut1 R155C iPSC line	Ludtmann et al.^44^ (Wray lab)	N/A
Human: VCPmut2 R155C iPSC line	Ludtmann et al.^44^ (Wray lab)	N/A
Human: VCPmut3 R191Q iPSC line	Ludtmann et al.^44^ (Wray lab)	N/A
Human: VCPmut4 R191Q iPSC line	Ludtmann et al.^44^ (Wray lab)	N/A
Human: TARDBPmut1 G298S iPSC line	Cedars-Sinai	CS47iALS-TDPnxx
Human: TARDBPmut2 G298S iPSC line	Target ALS	TALSTDP-47.10; RRID: CVCL_FA03

**Oligonucleotides**		

ASO-OGT sequence: GTGGCAGTTACAAACCCGTTACCAT	Parra et al.^29^	N/A
ASO-NC sequence: TTAGTTTAATCACGCTCG	This paper	N/A
Primer: *OGT* IRT Forward: GAAGCCAAGGTAGGTGTTTGA	Tyzack et al.^27^	N/A
Primer: *OGT* IRT Reverse: AAGAGTTGAAGACTTGGCAAA	Tyzack et al.^27^	N/A
Primer: *OGT* PCT Forward: TTCGCAGTGACCTGGGGAAC	This paper	N/A
Primer: *OGT* PCT Reverse: CACAGCCAAGATTACTCCAAGC	This paper	N/A
Primer: *GAPDH* Forward: ATGACATCAAGAAGGTGGTG	This paper	N/A
Primer: *GAPDH* Reverse: CATACCAGGAAATGAGCTTG	This paper	N/A

**Software and algorithms**		

Image Lab v6	Bio-Rad	
GraphPad Prism v11	Dotmatics	https://www.graphpad.com/
R Project for Statistical Computing v4	CRAN	RRID: SCR_001905
STAR v2.7	RNA-STAR	RRID:SCR_004463
Cutadapt	Martin^45^	RRID:SCR_011841
FlowJo v11	Waters Biosciences	https://www.flowjo.com/
Harmony High-Content Imaging and Analysis Software v5	Revvity	Cat#HH17000019
CellProfiler v4	Broad Institute	https://cellprofiler.org/

### Experimental model and study participant details

#### Compliance with ethical standards

For human iPSC work, informed consent was obtained from all donors. Experimental protocols were all conducted in accordance with the regulations and guidelines approved by the joint research ethics committee of UCLH’s National Hospital for Neurology and Neurosurgery and UCL Queen Square Institute of Neurology (09/0272).

#### hiPSC culture

hiPSCs were cultured in Essential 8 Medium (Gibco, A1517001) on Geltrex (Gibco, A1413302) and were passaged using UltraPure EDTA (Invitrogen, 15575020). All cell cultures were maintained at 37°C and 5% carbon dioxide. Cells used in this study were routinely confirmed to be mycoplasma-negative and authenticated by STR profiling through the Francis Crick Institute Cell Science facility. Cell lines from healthy donors and ALS patients were selected with consideration of donor sex where possible, to minimize potential sex-related differences. Details of the lines used in this study are provided in Table S1.

#### Differentiation of hiPSCs to motor neurons

Generation of hiPSC-MNs was performed using a previously described protocol.^46^ Briefly, neural induction was carried out in a chemically defined medium containing DMEM/F12 (Gibco, 31330038), Neurobasal (Gibco, 12348017), *N*-2 supplement (Gibco, 17502001), B27 supplement (Gibco, 12587001), GlutaMAX supplement (Thermo Scientific, 35050038), MEM non-essential amino acids (Gibco, 11140050), β-mercaptoethanol (Gibco, 21985023), and insulin (Sigma-Aldrich, I9278), supplemented with the small molecules: 1 μM dorsomorphin (Tocris, 3093), 2 μM SB431542 (Tocris, 1614), and 3.3 μM CHIR99021 (Tocris, 4423) for 7 days. After neural induction, neural precursors were patterned for 7 days with 0.5 μM retinoic acid (Sigma-Aldrich, R2625) and 1 μM purmorphamine (Sigma-Aldrich, 540220), followed by a 4-day culture with 0.1 μM purmorphamine. Motor neurons were terminally differentiated in 0.1 μM compound E (Tocris, 6476) for 6 days.

### Method details

#### Antisense oligonucleotides (ASOs) treatment

Phosphorothioate backbone, 2′ methoxyethyl-modified 25 nucleotide ASO was designed to target the *OGT* gene and shift RNA processing in favor of better intron removal through blocking a deep intronic cryptic splice site, without recruitment of RNase H. A non-human targeting ASO with the same modification was used as negative control (ASO-NC). ASO-OGT sequence was previously characterised in ref (5′- gtggcagttacaaacccgttaccat-3′).^29^ ASOs were dissolved in sterile nuclease-free H_2_O. Lipofectamine RNAiMAX (Invitrogen, 13778150) was applied to transfect ASOs with indicated concentrations in motor neuron progenitor cells according to the manufacturer’s guidelines. Medium was changed overnight after transfection for the neuronal terminal differentiation.

#### OGA inhibitor treatment

10 μM Thiamet G (TMG, Sigma-Aldrich, SML0244), as described in Zhao et al.,^26^ was added to day-4 terminally differentiated motor neuron culture for 48 h.

#### RNA extraction and qPCR

A Maxwell RSC simplyRNA Cells Kit (Promega, AS1390), including DNase treatment, was used for RNA extractions from hiPSC-MNs using the Maxwell RSC instrument. Nanodrop was used to assess RNA concentration and the 260/280 ratio. qPCR was performed on cDNA generated from 500 ng DNase-treated total RNA using SuperScript IV First-Strand Synthesis System (Invitrogen, 18091050), following the manufacturer’s instructions. qPCR reactions were performed in 10 μL volumes containing SYBR Green Mastermix (Applied Biosystems, 4309155) and 0.5 μM of the respective forward and reverse primers. Samples were amplified and analyzed using the QuantStudio 7 Flex Real-Time PCR System (Applied Biosystems). Cycling conditions were: 50°C for 2 min, 95°C for 2 min, followed by 40 cycles at 95°C for 15 s, then 60°C for 60 s. Samples were run in duplicate or triplicate and all programs contained a melt curve stage. RNA levels were calculated using the delta *C*_T_ method and normalized using the housekeeping gene *GAPDH*.

#### Protein quantification and western blotting

Cells were lysed in the lysis buffer containing 50 mM Tris-HCl pH 7.5 (Invitrogen, 15567027), 150 mM NaCl (Invitrogen, AM9760G), 1% NP-40 (Thermo Scientific, 85124), 1 mM EDTA (Invitrogen, AM9260G), and cOmplete Protease Inhibitor Cocktail (Roche, 4693159001). Protein concentration was determined using the Pierce BCA Protein Assay (Thermo Scientific, 23227). For Western blotting, approximately 10 μg of protein per sample was prepared with 10× NuPAGE reducing agent (Invitrogen, NP0009) and 4× NuPAGE LDS sample buffer (Invitrogen, NP0007) and heated at 70°C for 10 min. Proteins were loaded on NuPAGE 4–12% Bis-Tris gels (Invitrogen, WG1402BOX) in NuPAGE MOPS SDS Running Buffer (Invitrogen, NP0001). Following electrophoresis, proteins were transferred to nitrocellulose membranes using the iBLOT 2 transfer stack (Invitrogen, IB23001) and an iBLOT 2 Gel Transfer Device (Invitrogen). Following transfer, membranes were stained and imaged using the Revert Total Protein Stain Kit (LICORbio, 926–11016) and then blocked with 5% skim milk in 0.1% TBS-T for 1 h at room temperature. Membranes were subsequently incubated overnight at 4°C with the primary antibodies against OGT (1:500; Santa Cruz, SC-74546) or O-GlcNAc RL2 (1:1,000; Abcam, ab2739) diluted in 5% milk-TBS-T. Membranes were washed three times in TBS-T for 5 min each, followed by incubation for 1 h at room temperature with the corresponding HRP-conjugated secondary antibodies (1:5,000; Cytiva) in TBS-T. After three additional 5-min washes in TBS-T, signals were detected using ECL reagent (Cytiva, RPN2232). All membranes were imaged using a ChemiDoc MP imaging system (Bio-Rad). Band intensities were analyzed using Image Lab software (Bio-Rad).

#### Immunolabeling, imaging and analysis

Samples were fixed in 4% paraformaldehyde (Thermo Scientific, 043368.9M) diluted in PBS (Gibco, 10010015) and then blocked in 5% bovine serum albumin (BSA, Sigma-Aldrich, A7906) in PBS, containing 0.3% Triton X-100 (Thermo Scientific, 9002–93-1). Primary antibodies for PTBP1 (1:500; Proteintech, 67462-1-Ig or 12582-1-AP), U2AF65 (1:1000; Abcam, ab37530), hnRNPK (1:500; Santa Cruz, SC-28380), TDP-43 (1:500; Proteintech, 12892-1-AP), FUS (1:200; Santa Cruz, SC-47711), SFPQ (1:500; Abcam, ab38148), U2AF35 (1:500; Proteintech, 60289-1-Ig), beta III Tubulin (1:1000; Abcam, ab41489) were added overnight at 4°C in PBS containing 5% BSA. The next day, wells were washed with PBS and incubated with secondary antibodies (1:1,000; Invitrogen) and a 4′,6-diamidino-2-phenylindole (DAPI) nuclear stain in 5% BSA-PBS solution at room temperature for 1 h. Images were acquired in 96-well PhenoPlates (Revvity) using the Opera Phenix High-Content Screening System or on coverslips by Zeiss 880 Airyscan Microscope with 40× objective as confocal z-stacks and were processed as maximum projection. For each well in the PhenoPlate, ≥6 fields were acquired and cell density was measured as a quality control metric. Wells with sparse (<30) or excess (>300 × the number of fields) nuclei objects were excluded from the high-throughput analysis.

Image analysis was performed using CellProfiler or Harmony High-Content Analysis Software (Revvity), as described in Harley et al.^47^ Nuclei were identified and segmented based on DAPI staining to generate nuclear masks. The cytoplasmic region was defined by applying a proximal ring expansion from the nuclear boundary to capture the cytoplasm associated with each individual nucleus while minimizing overlap with neighboring cells. Mean fluorescence intensities for each RNA binding protein channel for each cell were then quantified within the nuclear and cytoplasmic compartments. Background signal was subtracted using measurements from cell-free regions within each field of view and no-primary-antibody negative control was used to account for non-specific fluorescence. Thresholds for object identification and signal detection were kept consistent across all conditions within each experiment to ensure comparability. A ratio of the nuclear to cytoplasmic average intensity was calculated per cell. An average of all fields of view in each coverslip/well was calculated. Within each experimental repeat, values for individual cell lines were calculated relative to the mean of the corresponding ASO-NC or DMSO-treated group. Values from multiple experimental repeats were subsequently averaged for each cell line, such that each biological cell line contributed one value to the statistical analysis.

#### Mapping of CLIP data

The human RNA-binding protein CLIP libraries were downloaded from ENCODE^31^^,^^32^ and analysis was conducted using a previously described pipeline.^27^ Briefly, adapter sequences were removed using Cutadapt,^45^ and reads shorter than 18 nucleotides were excluded before mapping. The remaining reads were aligned to UCSC hg19/GRCh37 genome assembly using STAR (RNA-STAR). Only uniquely mapped reads were used for subsequent analysis. Protein binding enrichment was quantified by comparing the number of mapped reads within the retained intron with the average number of reads mapped to non-retained intronic regions.

### Quantification and statistical analysis

Data analysis and associated graph generation were conducted using R or GraphPad Prism. Statistical tests used for each analytical or experimental dataset are reported within the figure legends, alongside the numbers of biological cell lines and experimental repeats. The choice between parametric and non-parametric tests was determined by the outcome of the Shapiro-Wilk normality test on the difference scores: parametric tests were used when normality was confirmed (*p*-value >=0.05), and non-parametric tests were applied otherwise. Statistical significance was accepted at *p*-value <0.05 and displayed as the actual value.
