# Supplementary material for: OGT regulates RNA-binding protein nucleocytoplasmic partitioning, but this fails in amyotrophic lateral sclerosis
Source: iScience. 2026 Oct 8;29(11):117807. doi: 10.1016/j.isci.2026.117807 (PMC13676730; doi:10.1016/j.isci.2026.117807)
Supplement: Supplementary file 1 [file mmc1.pdf]

iScience, Volume *29*

Supplemental information

OGT regulates RNA-binding protein nucleocytoplasmic partitioning, but this fails in amyotrophic lateral sclerosis

Yiran Wang, Lisa Fournier, Benjamin Wymann, Kevin Talbot, Raphaëlle Luisier, and Rickie Patani

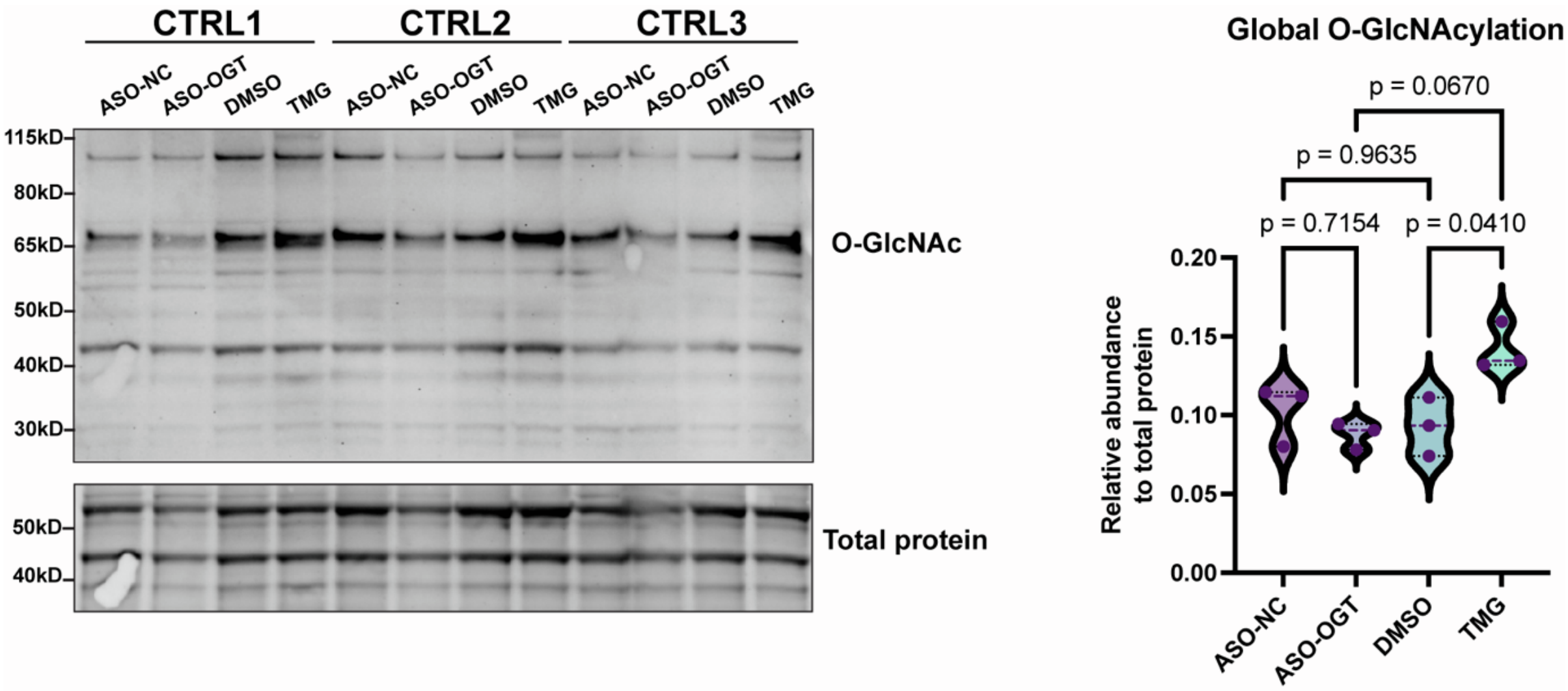

Supplementary Figure 1. The effect of ASO-OGT and TMG on global O-GlcNAcylation in hiPSC-derived motor neurons. Western blotting and quantification of hiPSC-MNs treated with ASO-OGT and TMG showing a significant increase of global O-GlcNAc modification induced by TMG treatment. Data are presented as violin plots. P-values from RM one-way ANOVA test and Tukey post-hoc multiple comparisons following the Shapiro-Wilk normality test, n=3 hiPSC-MN lines.

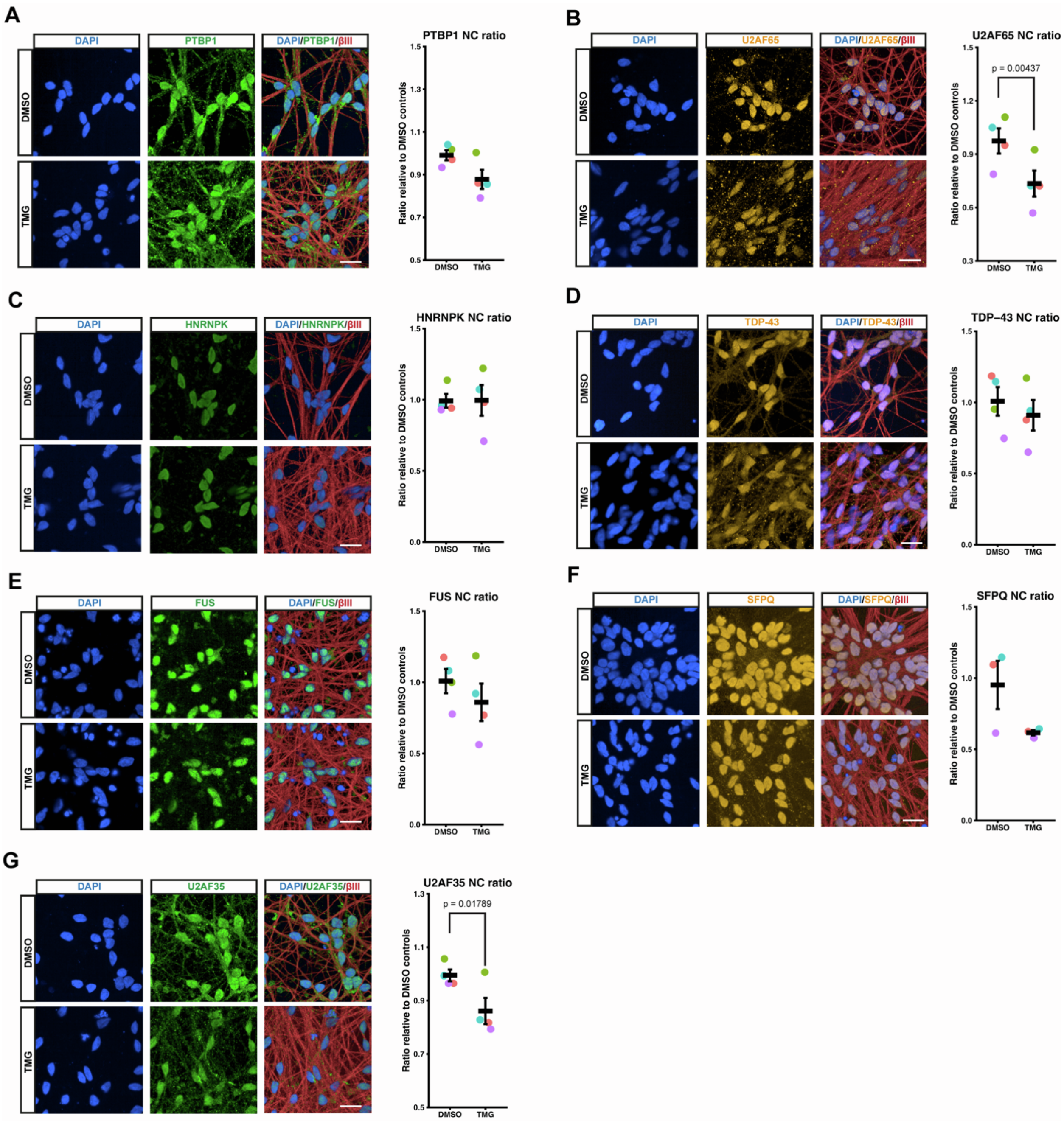

Supplemental Figure 2. RNA binding protein localization after OGA inhibition in ALS-*VCP* mutant hiPSC-derived motor neurons. Nucleocytoplasmic localization assay for RBPs PTBP1 (**A**), U2AF65 (**B**), hnRNPK (**C**), TDP-43 (**D**), FUS (**E**), SFPQ (**F**), and U2AF35 (**G**). n=3-4 ALS-*VCP* mutant lines, across two to four independent experimental repeats. Each point represents the mean for a cell line across independent experimental repeats, calculated relative to the mean of DMSO controls within each repeats (VCPmut1, orange; VCPmut2, green; VCPmut3, blue; VCPmut4, purple). P-values from the paired two-tailed t-test or Wilcoxon Signed-Rank test following the Shapiro-Wilk normality test. Data are presented as scatter plots with mean ± SEM. Scale bar = 20 µm.

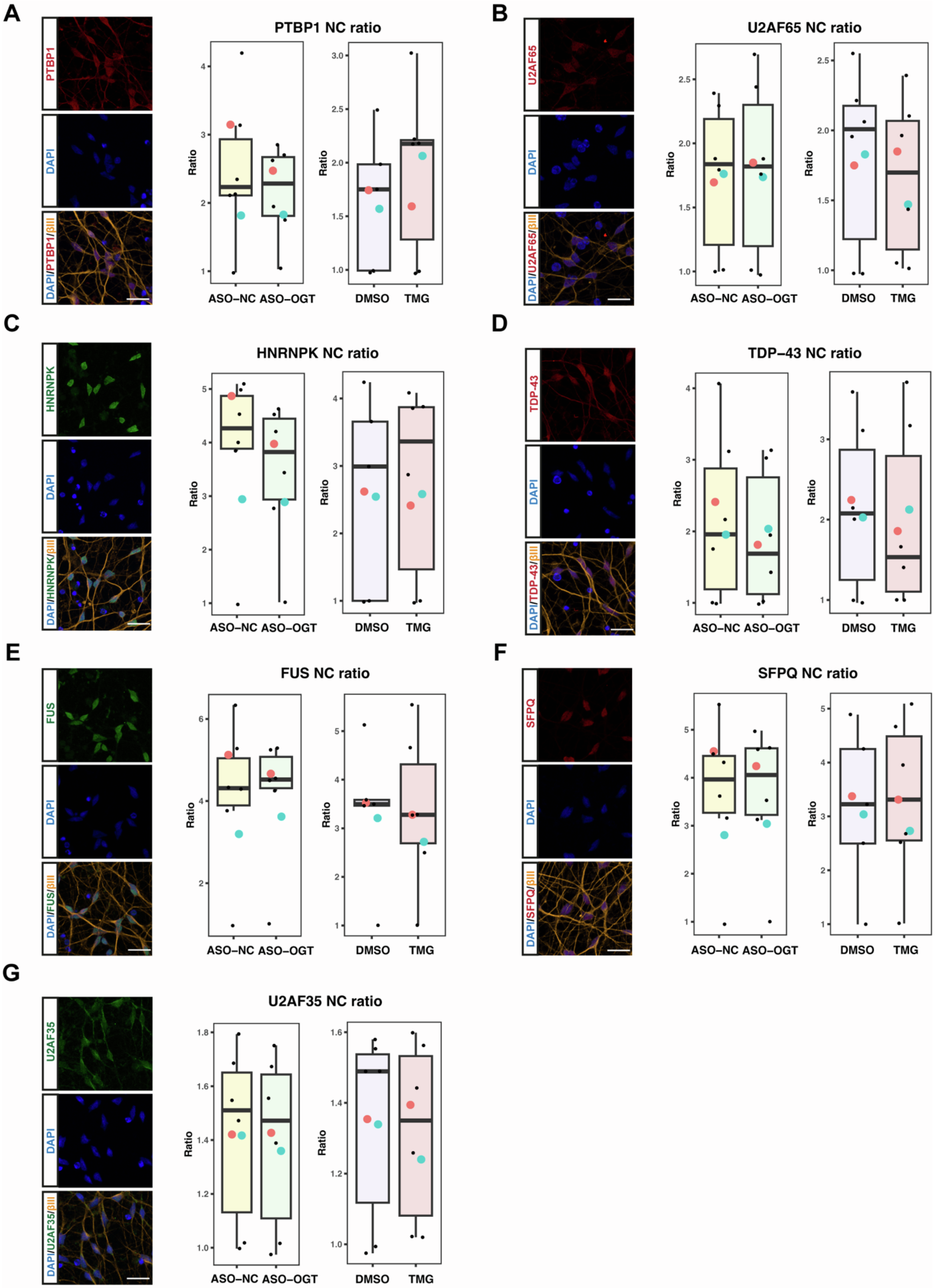
A
PTBP1
DAPI
DAPI/PTBP1/βIII
PTBP1 NC ratio
Ratio
ASO−NC
ASO−OGT
DMSO
TMG
B
U2AF65
DAPI
DAPI/U2AF65/βIII
U2AF65 NC ratio
Ratio
ASO−NC
ASO−OGT
DMSO
TMG
C
HNRNPK
DAPI
DAPI/HNRNPK/βIII
HNRNPK NC ratio
Ratio
ASO−NC
ASO−OGT
DMSO
TMG
D
TDP-43
DAPI
DAPI/TDP-43/βIII
TDP−43 NC ratio
Ratio
ASO−NC
ASO−OGT
DMSO
TMG
E
FUS
DAPI
DAPI/FUS/βIII
FUS NC ratio
Ratio
ASO−NC
ASO−OGT
DMSO
TMG
F
SFPQ
DAPI
DAPI/SFPQ/βIII
SFPQ NC ratio
Ratio
ASO−NC
ASO−OGT
DMSO
TMG
G
U2AF35
DAPI
DAPI/U2AF35/βIII
U2AF35 NC ratio
Ratio
ASO−NC
ASO−OGT
DMSO
TMG

Supplemental Figure 3. RNA binding protein localization after ASO treatment targeting *OGT* retained intron 4 or OGA inhibition in ALS-*TARDBP* mutant hiPSC-derived motor neurons. Nucleocytoplasmic localization assay for RBPs PTBP1 (**A**), U2AF65 (**B**), hnRNPK (**C**), TDP-43 (**D**), FUS (**E**), SFPQ (**F**), and U2AF35 (**G**). Two ALS-*TARDBP* mutant iPSC-MN lines, across three independent experimental repeats, were used for analysis. The mean ratio for each line across all repeats is highlighted in colour (TDP43mut1, orange; TDP43mut2, blue). Statistical analysis was performed using a linear model with treatment as the effect of interest and cell line × experimental repeat as a blocking factor to account for matched observations within each cell line and repeat; no significant treatment effect was detected. Data are presented as boxplots. Scale bar = 20 μm.

Supplemental Table 1. Human cell line information

Cell line	Mutation	Sex	Age	Source	Catalogue ID
CTRL1	Healthy	Female	Fetal	Thermo Fisher Scientific	A18945
CTRL2	Healthy	Female	51	Ludtmann et al. [1] (Wray lab)	N/A
CTRL3	Healthy	Male	51	Cedars-Sinai	CS0002iCTR-nxx
CTRL4	Healthy	Male	Fetal	NIH CRM	CRMi003-A
VCPmut1	*VCP* R155C	Female	43	Ludtmann et al. [1] (Wray lab)	N/A
VCPmut2	*VCP* R155C	Female	43	Ludtmann et al. [1] (Wray lab)	N/A
VCPmut3	*VCP* R191Q	Male	42	Ludtmann et al. [1] (Wray lab)	N/A
VCPmut4	*VCP* R191Q	Male	42	Ludtmann et al. [1] (Wray lab)	N/A
TDP43mut1	*TARDBP* G298S	Male	64	Cedars-Sinai	CS47iALS-TDPnxx
TDP43mut2	*TARDBP* G298S	Male	43	Target ALS	TALSTDP-47.10

Reference

[1] Ludtmann, M.H.R., Arber, C., Bartolome, F., de Vicente, M., Preza, E., Carro, E., Houlden, H., Gandhi, S., Wray, S., and Abramov, A.Y. (2017). Mutations in valosin-containing protein (VCP) decrease ADP/ATP translocation across the mitochondrial membrane and impair energy metabolism in human neurons. J. Biol. Chem. *292*, 8907–8917.
